# Early and Late Onset Neonatal Sepsis: Epidemiology and Effectiveness of Empirical Antibacterial Therapy in a III Level Neonatal Intensive Care Unit

**DOI:** 10.3390/antibiotics11020284

**Published:** 2022-02-21

**Authors:** Marcello Mariani, Alessandro Parodi, Diego Minghetti, Luca A. Ramenghi, Candida Palmero, Elisabetta Ugolotti, Chiara Medici, Carolina Saffioti, Elio Castagnola

**Affiliations:** 1 Department of Neuroscience, Rehabilitation, Ophthalmology, Genetics, Maternal and Child Health (DINOGMI), University of Genoa, Largo P. Daneo, 3, 16132 Genoa, Italy; 2 IRCCS Istituto Giannina Gaslini, Children’s Hospital, 5, 16147 Genoa, Italy; alessandroparodi@gaslini.org (A.P.); diegominghetti@gaslini.org (D.M.); lucaramenghi@gaslini.org (L.A.R.); candidapalmero@gaslini.org (C.P.); elisabettaugolotti@gaslini.org (E.U.); chiaramedici@gaslini.org (C.M.); carolinasaffioti@gaslini.org (C.S.); eliocastagnola@gaslini.org (E.C.)

**Keywords:** EOS, LOS, sepsis, antibiotic therapy

## Abstract

Bloodstream infections play an important role in neonatal morbidity and mortality. In this study, we retrospectively analyzed etiology and antibiotic resistance profiles of bacteria isolated from blood or Cerebro Spinal Fluid (CSF) cultures to evaluate the appropriateness of initial empirical therapy of neonatal sepsis. Methods: microbiological data from patients admitted to Neonatal Intensive Care Unit (NICU), from January 2005 to October 2018, were anonymously extracted from the Laboratory of Microbiology database. According to the neonatal sepsis definition for patients admitted to NICU, positive cultures obtained within the first 72 h of life were labeled as Early Onset Sepsis (EOS); and Late Onset Sepsis (LOS) for those obtained later. Results: 859 bacterial strains, 846 from blood and 13 from CSF, were detected in 611 neonates. In EOS, 75 blood cultures were found: 61 yielded Gram-positives and 14 Gram-negatives. Coagulase Negative Staphylococci (CoNS) represented the majority (52% *n* = 39). *Streptococcus agalactiae* and *Escherichia coli* were both isolated in 8% (*n* = 6) of cases. 784 strains were isolated in LOS: 686 (87%) Gram-positives and 98 (13%) Gram-negatives. CoNS represented most pathogens (*n* = 560, 71.4%) followed by *Staphylococcus aureus* (*n* = 57, 7.3%) and *Enterococcus faecalis* (*n* = 33, 4.2%). Ampicillin/gentamicin therapy resulted effective in 15/20 (75%) of EOS isolates. Internal protocol for LOS initial empirical therapy, calling for piperacillin/tazobactam and vancomycin resulted effective in 98.5% (734/745) of LOS strains. Conclusions: knowledge of local epidemiology of resistant pathogens, both in EOS and LOS, is fundamental to set up an effective empirical therapy in NICU. Aminoglycosides were fundamental in EOS. On the other side, LOS empirical therapy with vancomycin is sustained by the observation of 38% of methicillin resistance among *S. aureus* and about 95% in CoNS.

## 1. Introduction

Bloodstream infections play an important role in neonatal morbidity and mortality and are a leading cause of long-term sequelae [1,2]. Early-onset sepsis (EOS) is defined as the presence of bacteremia or meningitis in the first 72 h of life in neonates admitted to a neonatal intensive care unit (NICU) and in the first 7 days of life in full-term newborns [3]. Late-onset sepsis (LOS) is defined after 72 h in neonates admitted to NICU and after 7 days in full-term newborns. Distinction between EOS and LOS on a chronological criterion is used to remark differences on bacterial epidemiology and has an impact on empirical antibiotic therapy. An association of a beta lactam (ampicillin) plus an aminoglycoside (gentamicin) is usually recommended for EOS by international guidelines to obtain coverage against *E. coli* and *S. agalactiae* (group B Streptococcus, GBS) [3,4]. LOS therapy should include beta lactams mainly for their central nervous system (CNS) penetration [5] and a glycopeptide for the major presence of Gram-positive pathogens such as *Staphylococcus aureus* and methicillin-resistant coagulase-negative staphylococci (CoNS) responsible for 48–55% of sepsis in very low birthweight infants (VLBW) [6]. Despite these indications, it is mandatory to adapt therapeutic choices on local epidemiology since LOS is usually healthcare-related [7,8,9], being invasive procedures, such as central vascular accesses positioning and use, or mechanical ventilation, well known risk factors for LOS [10,11]. Adapting antibiotic therapy protocols to local bacterial ecology and resistance profiles can contribute to increasing therapy effectiveness and reducing adverse effects [12,13,14,15,16].

In this study, we retrospectively analyzed etiology and antibiotic resistance profiles of bacteria isolated from blood or CSF cultures in order to evaluate the appropriateness of initial empirical therapy for EOS and LOS in a tertiary care NICU.

## 2. Materials and Methods

The IRCCS *Istituto Giannina Gaslini* (IGG) is a tertiary-care pediatric hospital in Genoa, northern Italy, serving both local urban area and Liguria region and representing an important referral hospital for Italy and many foreign countries. Microbiological data from patients admitted to NICU, from January 2005 to October 2018, were anonymously extracted from the Laboratory of Microbiology database, according to the IGG data protection policy based on European Union Data protection rules (https://ec.europa.eu/commission/priorities/justice-and-fundamental-rights/data-protection/2018-reform-eu-data-protection-rules_en, accessed on 31 December 2018). For study purposes, only cultures from blood or cerebrospinal fluid (CSF) were eligible for analysis. According to neonatal sepsis definition for NICU patients [3], positive cultures obtained within the first 72 h of life were labeled as EOS; and LOS those obtained later.

Antibiotic resistance or sensitivity was tested and interpreted according to EUCAST clinical breakpoint criteria [17]. Since these definitions could change, during the study period, pathogens were divided into resistant (R) or sensitive (S), without any considerations over the minimal inhibitory concentration values. As IGG internal protocol foresees use of antibiotics at maximal dosages, intermediate strains (I) were considered as “susceptible, increased exposure”, according to 2020 EUCAST definitions.

To evaluate case series complexity, a total count of the following ICD9-CM discharge diagnosis was performed searching for the following codes:

- 7650 (subcodes 76501, 76502, 76503, 76504, 76505) “Disorders relating to extreme immaturity of infant”.
- 7651 (subcodes 76511, 76512, 76513, 76514, 76515) “Disorders relating to other preterm infants”.
- 7652 (subcodes 76521, 76522, 76523, 76524, 76525, 76526, 76527) “Weeks of gestation”.

Patients were considered “premature” if born before 34 weeks of gestation and VLBW if weighing <1500 g at birth. Since the study was based on the data from the Laboratory of Microbiology no other demographic or clinical information could be retrieved.

### 2.1. Standard of Care

EOS treatment protocol included ampicillin plus gentamicin [18]. Ampicillin plus cefotaxime were administered for suspected sepsis in patients treated with therapeutic hypothermia [19,20]. LOS treatment included a combination of vancomycin plus piperacillin-tazobactam. All patients weighing less than 1500 g underwent fluconazole prophylaxis for invasive candidiasis [21]. 

### 2.2. Statistical Analysis

Categorical variables were reported as total numbers and proportions (percentages, %). Infection epidemiology was analyzed by calculating the rate of episodes, i.e., the number of episodes observed in one year divided by the total number of admissions or hospitalization days in the same period and normalized to 1000 observations (episodes/1000 admissions or /1000 hospitalization-days). To measure the association between year and isolation rates Pearson correlation r-coefficient and Spearman’s rho were used for normally and non-normally distributed variables, respectively. Shapiro–Wilk test for normality was used. Chi-square and Mann–Whitney U tests were used to compare non-normally distributed samples by grouping variables of EOS and LOS using Jamovi, an open source R graphical frontend [22,23].

## 3. Results

During the study period 859 bacterial strains, 846 from blood and 13 from CSF, were detected in 611 neonates. All positive CSF were found in LOS (*p* < 0.001). Among these 611 patients, 438 (71.7%) had only one positive culture during the entire hospital stay while 173 (28.3%) had more than one, for a total of 421 isolated strains. In 57/421 of these episodes (13.5%) a second blood culture followed the first within 72 h and in 10/57 (17.5%) persistence of the same pathogen was documented. The overall isolates rate was 7/1000 patient days and 282/1000 hospitalizations. No correlations were observed between study year and infection rates for 1000 patient days or 1000 hospital admissions. Population data are summarized in Table 1.

The analysis of ICD codes (available since 2009) showed a positive correlation between study year and rate of preterm newborns admissions (Spearman rho = 0.753, *p* = 0.002). Similarly, a second positive correlation was observed between study year and VLBW neonate admissions (Spearman rho = 0.819, *p* = < 001).

### 3.1. Pathogens Involved in EOS and LOS

A total of 75 positive blood cultures were observed in EOS, 61 yielded Gram-positives and 14 Gram-negatives. CoNS represented the majority (52% *n* = 39) of isolated pathogens. *Streptococcus agalactiae* and *Escherichia coli* were both isolated in 8% (*n* = 6) of cases. Other common EOS pathogens were *Staphylococcus aureus* (*n* = 4, 5.3%) and *Listeria monocytogenes* (*n* = 2, 2.7%).

A total of 784 strains were isolated in LOS: 686 (87%) Gram-positives and 98 (13%) Gram-negatives. In this case, CoNS also represented the most frequently isolated pathogens (*n* = 560, 71.4%) followed by *Staphylococcus aureus* (*n* = 57, 7.3%) and *Enterococcus faecalis* (*n* = 33, 4.2%). Gram-negatives were mainly represented by *Escherichia coli* (*n* = 33, 4.2%) and *Klebsiella pneumoniae* (*n* = 20, 2.6%). Microbiological data, including all pathogens isolated in EOS and LOS, are summarized in Supplementary Tables S1 and S2.

### 3.2. Antibiotic Susceptibility

**Figure 1:**
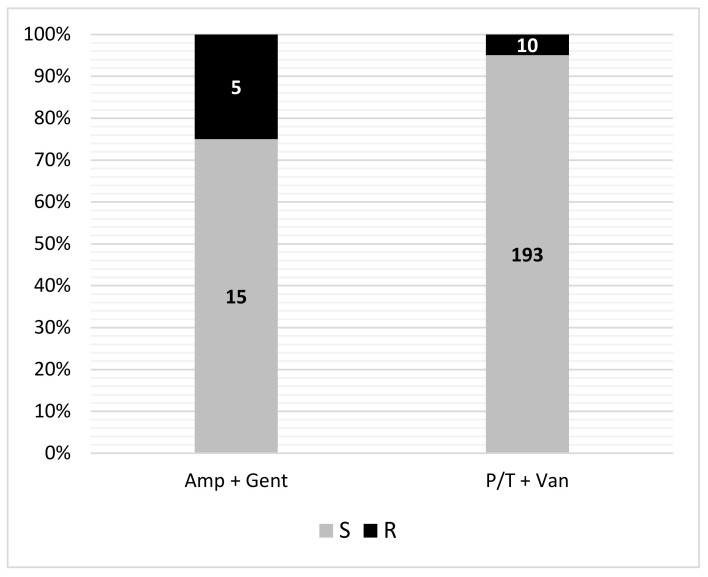
Sensitivity of isolates to LOS and EOS empiric therapy. S = documented sensitivity to at least one antibiotic. R = documented resistance to both antibiotics or documented resistance to one with the second not tested. CoNS were excluded from this analysis.

After CoNS exclusion from this analysis, ampicillin plus gentamicin combination therapy resulted effective in 15/20 (75%) of tested EOS isolates (Figure 1).

Resistance to one molecule with the second not tested was documented in 3/20 (15%) and concomitant resistance to both antibiotics in 2/20 (10%) of tested strains. Noteworthy, for *S. agalactiae* ampicillin was not directly tested but sensitivity inferred from penicillin susceptibility, increasing therapy overall efficacy to 81% (*n* = 21/26).

Table 2 summarizes antibiotic sensitivity for pathogens with more than 10 isolates. In EOS, *E. coli* resistance to ampicillin was 100% and 16% to gentamycin. Methicillin resistance was observed in 25% of *S. aureus* strains and in 91% of CoNS. No ampicillin resistance was observed in *E. faecalis* or *S. agalactiae*.

Regarding LOS, the combination of piperacillin/tazobactam and vancomycin resulted effective in 98.5% (734/745) of tested strains and in 95% (193/203) after CoNS exclusion (Figure 1). Among Gram-positives 87.5% (*n* = 686/784) resulted methicillin-resistant, while piperacillin–tazobactam resistance among Gram-negatives was 11.4% (*n* = 10/88). In detail, methicillin resistance was observed in 30% of *S. aureus*, and 94.7% of CoNS. Vancomycin resistance was observed in a single CoNS strain. Among Gram-negatives 3.4% of *E. coli*, 26.1% of *K. pneumoniae,* and 8.3% of *K. oxytoca* were resistant to piperacillin/tazobactam. No carbapenem-resistant strains were isolated. Proportions of infection by antibiotic-resistant strains are shown in Supplementary Table S3.

## 4. Discussion

This study provides an overview of epidemiology of bacteria isolated from blood or CSF cultures (invasive infections) in a large Italian tertiary care NICU. In spite of an increasing cases complexity, we did not observe an increase of invasive infections.

Most frequently EOS isolated strains were “classical” neonatal pathogens as *E. coli* and *S. agalactiae*, although a non-negligible presence of *S. aureus* was detected, while only 2 cases due to *Listeria monocytogenes* were recorded in 13 years of study. Looking to antibiotic susceptibility in EOS, it emerged that empirical therapy with ampicillin and gentamicin combination was effective on about 75% of tested bacteria. This percentage is probably underestimated as we excluded *S. agalactiae* strains as ampicillin was not directly tested, but sensitivity inferred from penicillin [17]. Noteworthy, in EOS *E. coli* was isolated with the same frequency of *S. agalactiae*, but ampicillin resistance was total for the Gram-negative. The high rate of ampicillin resistance among Gram-negatives makes the association with an aminoglycoside, essential for obtaining a high coverage rate of EOS main pathogens. Therapy could be de-escalated after bacterial identification and antibiotic susceptibility tests. This observation of a non-negligible proportion of strains resistant to drugs usually administered for EOS, could be at least partially due to resistant pathogens early acquired after delivery and already present at time of admission in NICU [24]. This aspect must be carefully monitored in the future.

LOS isolates were mainly represented by Gram-positives, mainly CoNS and *S. aureus*. An important number of Gram negatives as *E. coli*, *K. pneumoniae, K. oxytoca,* and *Enterobacter* were also identified. Empirical therapy with piperacillin/tazobactam plus vancomycin was effective on 98.5% of LOS isolates. Only a small number of *E. coli* and *Klebsiella* strains showed piperacillin resistance. In these cases, meropenem was 100% effective as no carbapenem resistant Gram-negatives was isolated. Noteworthy, piperacillin would be useful also against *S. agalactiae* than can be isolated in LOS [25], and not only in EOS.

In our center, the use of vancomycin in LOS empirical therapy is sustained by the observation of 38% of methicillin resistance among *S. aureus* and about 95% in CoNS, which represented more than 70% of LOS isolates. Even if a clear attribution to CoNS of an invasive infection in newborns could be difficult, in our opinion it is still important to consider also CoNS local susceptibility for LOS empirical therapy since in “fragile” patients (such VLBW and/or preterm newborns), even a single blood culture positive for these pathogens could be the beacon of an invasive disease [26].

Our study has limitations: firstly, the retrospective nature of our analysis, although supported by a large sample size, could have been influenced by external independent factors, such as adherence to treatment protocols or hand hygiene procedures. Secondly, resistance pattern changes could not only be secondary to antibiotics use (or misuse) but, especially in a national referral hospital, such as IGG, be influenced by patients coming from other centers. Despite these caveats, we believe that knowledge of epidemiology of resistant pathogens both in EOS and LOS is fundamental to set up an effective empirical therapy in NICU.

## Figures and Tables

**Table 1:** Summary of epidemiological data.

Year	Total Admissions	Total Patient Days	<34 Weeks		<1500 g		Total Isolates			EOS	LOS	Gram-Positives	Gram-Negatives
	*n*		*n*	Episodes/ 1000 admissions	*n*	Episodes/ 1000 admissions	*n*	Episodes/ 1000 patient days	Episodes/ 1000 admissions	*n*	*n*	*n*	*n*
2005	67	5295	-	0		0	21	4	313	5	16	18	3
2006	277	5348	-	0		0	75	14	271	12	63	67	8
2007	197	4431	-	0		0	45	10	228	6	39	40	5
2008	90	2348	-	0	16	178	35	15	389	4	31	27	8
2009	234	5807	62	265	41	175	65	11	278	5	60	54	11
2010	258	5625	57	221	59	229	63	11	244	4	59	59	4
2011	230	16777	90	391	50	217	90	5	391	0	90	80	10
2012	166	18875	29	175	55	331	137	7	825	4	133	119	18
2013	191	18426	25	131	50	262	62	3	325	5	57	54	8
2014	253	8465	15	59	99	391	54	6	213	8	46	49	5
2015	280	8840	32	114	104	371	61	7	218	1	60	53	8
2016	273	7973	166	608	88	322	53	7	194	4	49	44	9
2017	312	7538	136	436	69	221	42	6	135	9	33	37	5
2018	213	6759	138	648	79	371	56	8	263	8	48	46	10
TOTAL	3041	122507	750	247		0	859	7	282	75	784	747	112

**Table 2:** Antibiotic resistance profiles for pathogens with > 10 isolates. EOS: early-onset sepsis; LOS: late-onset sepsis. R = resistant.

Pathogen	% Ampicillin-R		% Gentamycin-R		% Methicillin-R		% Piperacillin/Tazobactam-R		% Ceftazidime-R		% Meropenem-R		% Vancomycin-R	
	EOS	LOS	EOS	LOS	EOS	LOS	EOS	LOS	EOS	LOS	EOS	LOS	EOS	LOS
*S. aureus* (*n* = 61)	50	85	25	38	25	30	-	-	-	-	-	-	0	0
	(1/2)	(35/41)	(1/4)	(21/55)	(1/4)	(17/56)							(0/4)	(0/56)
CoNS (*n* = 599)	93	99.8	66	93	74	95	-	-	-	-	-	-	0	0.2
	(28/30)	(462/463)	(25/38)	(501/536)	(28/38)	(502/530)							(0/38)	(1/542)
*E. faecalis* (*n* = 34)	0	0	0	0	-	-	-	-	-	-	-	-	0	0
	(0/1)	(0/33)	(0/1)	(0/6)									(0/1)	(0/33)
*E. coli* (*n* = 39)	100	82	17	10	-	-	0	3.4	17	0	0	0	-	-
	(3/3)	(18/22)	(1/6)	(3/29)			(0/6)	(1/29)	(1/6)	(0/29)	(0/6)	(0/29)		
*K. pneumoniae* (*n* = 20)	-	100	-	16	-	-	-	26	-	28	-	0	-	-
		(14/14)		(3/19)				(5/19)		(5/19)		(0/19)		
*S. agalactiae* (*n* = 23)	0	0	-	-	-	-	-	-	-	-	0	0	0	0
	(0/1)	(0/1)									(0/1)	(0/1)	(0/4)	(0/8)
*K. oxytoca* (*n* = 15)	-	100	-	0	-	-	-	8	-	0	-	0	-	-
		(10/10)		(0/12)				(1/12)		(0/12)		(0/12)		
*E. cloacae* (*n* = 11)	-	100	-	0	-	-	-	18	-	18	-	0	-	-
		(9/9)		(0/11)				(2/11)		(2/11)		(0/11)		

Proportions (%) are numbers of resistant strains over number of tested strains.

## Data Availability

Data are contained within the article or Supplementary Materials. They are stored in a IGG database and can be available on request from the corresponding author. The data are not publicly available due to personal data protection rules.

## Supplementary Materials

The following supporting information can be downloaded at: https://www.mdpi.com/article/10.3390/antibiotics11020284/s1. Table S1: yearly events number, and isolates strains stratified by EOS and LOS. Table S2: pathogens isolated in persistently positive blood cultures at 72 hours. Table S3: antibiotic susceptibility for Gram positives and Gram negatives with respective crude rates (*100 isolated strains).
