# Supplementary material for: Early and Late Onset Neonatal Sepsis: Epidemiology and Effectiveness of Empirical Antibacterial Therapy in a III Level Neonatal Intensive Care Unit
Source: Antibiotics (Basel). 2022 Feb 21;11(2):284. doi: 10.3390/antibiotics11020284 (PMC8868064; doi:10.3390/antibiotics11020284)
Supplement: Supplementary file 1 [file antibiotics-11-00284-s001.zip › antibiotics-1594788-supplementary.pdf]

antibiotics

MDPI

Supplementary File

Table S1. yearly events number, and isolates strains stratified by EOS and LOS.

Variable	Subvariable	Total	EOS		LOS		P value
			N	% on EOS	N	% on LOS	
**Year**	2005	21	5	6,7	16	2,0	0,67
	2006	75	12	16,0	63	8,0	
	2007	45	6	8,0	39	5,0	
	2008	35	4	5,3	31	4,0	
	2009	65	5	6,7	60	7,7	
	2010	63	4	5,3	59	7,5	
	2011	90	0	0,0	90	11,5	
	2012	137	4	5,3	133	17,0	
	2013	62	5	6,7	57	7,3	
	2014	54	8	10,7	46	5,9	
	2015	61	1	1,3	60	7,7	
	2016	53	4	5,3	49	6,3	
	2017	42	9	12,0	33	4,2	
	2018	56	8	10,7	48	6,1	
		859	75		784		
**Sample type**	Blood	846	75	100	771	98,3	**<0,001**
	CSF	13	0	0	13	1,7	
		859	75		784		
**Gram stain**	Positive	747	61	81,3	686	87,5	0,13
	Negative	112	14	18,7	98	12,5	
		859	75		784		
**Microbial species**	CoNS	599	39	52	560	71,4	**<0,001**
	Staphylococcus aureus	61	4	5,3	57	7,3	
	Enterococcus faecalis	34	1	1,3	33	4,2	
	Escherichia coli	39	6	8,0	33	4,2	
	Klebsiella pneumoniae	20			20	2,6	
	Streptococcus agalactiae	23	6	8,0	17	2,2	
	Klebsiella oxytoca	15			15	1,9	
	Enterobacter cloacae	11			11	1,4	
	Streptococcus viridans	6			6	0,8	
	Serratia marcescens	5			5	0,6	
	Enterobacter aerogenes	4			4	0,5	
	Streptococcus mitis	5	2	2,7	3	0,4	
	Enterococcus spp.	3	1	1,3	2	0,3	
	Micrococcus species	2			2	0,3	
	Micrococcus luteus	4	2	2,7	2	0,3	
	Bacillus coagulans	1			1	0,1	
	Morganella morganii	2	1	1,3	1	0,1	
	Pseudomonas aeruginosa	1			1	0,1	
	Enterococcus hirae	1			1	0,1	
	Abiotrophia species	1			1	0,1	
	Enterobacter spp.	1			1	0,1	
	Streptococcus bovis	1			1	0,1	

	Stenotrophomonas maltophilia	1			1	0,1
	Citrobacter spp.	1			1	0,1
	Klebsiella spp	1			1	0,1
	Citrobacter koseri	1			1	0,1
	Serratia plymuthica	1			1	0,1
	Enterococcus faecium	2	1	1,3	1	0,1
	Streptococcus sanguinis	2	1	1,3	1	0,1
	Haemophilus influenzae	2	2	2,7		
	Listeria monocytogenes	2	2	2,7		
	Corynebacterium species	2	2	2,7		
	Corynebacterium matruchotii	1	1	1,3		
	Streptococcus pneumoniae	1	1	1,3		
	Proteus mirabilis	1	1	1,3		
	Streptococcus vestibularis	1	1	1,3		
	Chryseobacterium indologenes	1	1	1,3		
		859	75		784	

Table S2.: pathogens isolated in persistently positive blood cultures at 72 hours.

Isolated Strain	Count	% Over Persistently Isolated Pathogens
Staphylococcus epidermidis	5	50
Escherichia coli	1	10
Staphylococcus haemolyticus	1	10
Klebsiella oxytoca	1	10
Staphylococcus warneri	1	10
Staphylococcus coagulase-negative	1	10

Table S3. antibiotic susceptibility for Gram positives and Gram negatives with respective crude rates (*100 isolated strains).

Year	Gram +	Gram -	G+ met R	G- amp R	G- pip/tazo R	G- genta R
**2005**	18	3	14 (78)	1 (33)	0	0
**2006**	67	8	46 (69)	5 (63)	0	0
**2007**	40	5	26 (65)	2 (40)	0	1 (20)
**2008**	27	8	21 (78)	6 (75)	0	0
**2009**	54	11	45 (83)	10 (91)	2 (18)	0
**2010**	59	4	52 (88)	3 (75)	0	0
**2011**	80	10	60 (75)	9 (90)	1 (10)	0
**2012**	119	18	93 (78)	16 (89)	3 (17)	1 (6)
**2013**	54	8	38 (70)	6 (75)	1 (13)	1 (13)
**2014**	49	5	36 (73)	4 (80)	0	1 (20)
**2015**	53	8	42 (79)	5 (63)	0	0
**2016**	44	9	25 (57)	1 (11)	3 (33)	3 (33)
**2017**	37	5	23 (62)	0	0	0
**2018**	46	10	29 (63)	0	1 (10)	1 (10)

numbers in parenthesis are the rate of resistant strains over 100 isolates of the same Gram stain group.
